# Facile construction of a DNA tetrahedron in unconventional ladder-like arrangements at room temperature†

**DOI:** 10.1039/c8na00323h

**Published:** 2018-12-27

**Authors:** Ziwen Dai, Hoi Man Leung, Qi Gao, Fei Wang, Sze Wing Wong, Ling Sum Liu, Yu Ju Au, King Wai Chiu Lai, Pik Kwan Lo

**Affiliations:** Department of Chemistry, City University of Hong Kong Tat Chee Avenue Kowloon Tong Hong Kong SAR peggylo@cityu.edu.hk; Department of Biomedical Engineering, City University of Hong Kong Tat Chee Avenue Kowloon Tong Hong Kong SAR; Key Laboratory of Biochip Technology, Biotech and Health Care, Shenzhen Research Institute of City University of Hong Kong Shenzhen 518057 China

## Abstract

A DNA tetrahedron as the most classical and simplest three-dimensional DNA nanostructure has been widely utilized in biomedicine and biosensing. However, the existing assembly approaches usually require harsh thermal annealing conditions, involve the formation of unwanted by-products, and have poor size control. Herein, a facile strategy to fabricate a discrete DNA tetrahedron as a single, thermodynamically stable product in a quantitative yield at room temperature is reported. This system does not require a DNA trigger or thermal annealing treatment to initiate self-assembly. This DNA tetrahedron was made of three chemically ligated triangular-shaped DNAs in unconventional ladder-like arrangements, with measured heights of ∼4.16 ± 0.04 nm, showing extra protections for enzymatic degradation in biological environment. They show substantial cellular uptake in different cell lines *via* temperature, energy-dependent and clathrin-mediated endocytosis pathways. These characteristics allow our DNA tetrahedron to be used as vehicles for the delivery of very small and temperature-sensitive cargos. This novel assembly strategy developed for DNA tetrahedra could potentially be extended to other highly complex polyhedra; this indicated its generalizability.

## Abstract 2

An isothermal assembly of DNA tetrahedron at room temperature in a quantitative yield.

## Graphical abstract

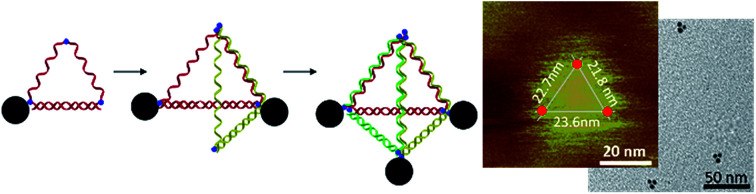

## Introduction

With the rapid development of DNA nanotechnology, various bottom-up self-assembly strategies have been utilized to construct DNA nanostructures with different shapes and sizes.^1,2^ These nanostructures have been used for *in vitro* applications such as nanotools for molecular biology, biosensors, and smart nanodevices.^3–5^ Due to their excellent biocompatibility and cellular permeability, they have become a promising platform for drug delivery applications.^6,7^ As the most classical and simplest three-dimensional (3D) structure, a DNA tetrahedron can be synthesized by mixing four single-stranded DNAs in one-pot after a quick thermal annealing process.^8^ The average hydrodynamic size of the tetrahedron constructed by Turberfield and his co-workers was ∼7–10 nm.^9^ Mao *et al.* subsequently adopted a tile-based hierarchical assembly strategy in which symmetric three-point-star motifs were assembled first and then connected with each other to form the desired tetrahedral structures or multi layered DNA cages after heating and cooling treatment.^10,11^ However, many of the biomedical applications for the DNA tetrahedron involve temperature-sensitive cargos; thus, systems that require harsh thermal annealing for their final assembly have great disadvantages.^12^ Alternatively, an origami-based assembly has been applied to hybridize the DNA scaffold and staple strands to result in planar triangular faces; this is followed by the closing of each of the faces to form a DNA tetrahedron.^13^ Due to the substantial number of strands used in this approach, this method suffers from high production cost and also results in a very large and rigid DNA tetrahedron,^14^ for example, cages with 54 nm edges and an internal cavity with a volume of 15 000 nm^3^.^15^ On the other hand, this origami approach is highly limited to the construction of very small sized 3D nanostructures. Moreover, origami structures with defects could not be suppressed; this led to further problems of separation and purification.^16^

To date, DNA tetrahedra have been extensively studied as potential nanocarriers for drug delivery due to their high cell permeability and biocompability.^17^ Recently, Yin's group developed a new synthetic strategy to grow a 3D wireframe DNA tetrahedron by ring forming reactions in the presence of an initiator.^18^ Kang *et al.* reported that the size of the tetrahedral nanocarrier could be a key point of importance in its cellular uptake and internalization with various endocytic vesicles. They found that smaller sized tetrahedra showed higher internalization in cancer cells within a few hours.^19^ Additionally, phagocytosis of DNA nanocarriers with small sizes can be circumvented to minimize inflammatory reactions and thus enhance the delivery efficiency to particular target tissues.^20^

**Scheme 1:**
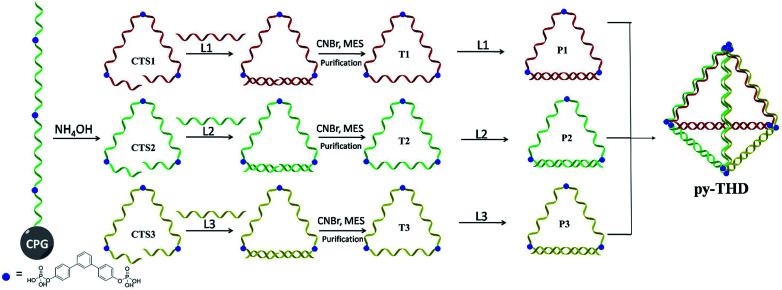
The assembling strategy for the construction of a discrete DNA tetrahedron.

To address the problems of the generation of unwanted by-products, poor size control, and harsh annealing conditions, development of a simple strategy to assemble discrete 3D DNA tetrahedra as a single product without thermal annealing is highly necessary and challenging. Herein, we report the rational design and a novel method to construct DNA tetrahedra either in a modular or one-pot self-assembly manner. In this study, six DNA building blocks, including three cyclic triangular-shaped DNA scaffolds with 15 nucleobases at each side and three 15-mer complementary strands, were mixed sequentially or randomly to assemble discrete DNA tetrahedra at room temperature with 100% yield (Scheme 1). The successful formation of these defect-free DNA tetrahedra was characterized by atomic force microscopy (AFM), transmission electron microscopy (TEM) and polyacrylamide gel electrophoresis (PAGE) analyses. This system does not require a DNA trigger or thermal annealing treatment to initiate the self-assembly and foster the dominant formation of the desired, lowest-energy product. This very small DNA tetrahedron exhibited extra protections for enzymatic degradation as compared to duplex DNAs and substantial cellular uptake in different cell lines within 12 h *via* temperature-, energy-dependent and clathrin-mediated endocytosis pathway. Contrary to previously studied DNA assemblies, the chemically ligated triangular-shaped DNAs facilitate the facile construction of a discrete tetrahedral DNA nanostructure at room temperature; thus, they can be used for the construction of highly complex polyhedra such as cube, pentagon, hexagon, *etc.* in an efficient way.

## Experimental

### Chemical ligation

The DNA strands CTS1, CTS2 and CTS3 used for chemical ligation were synthesized on 3′-phosphate-modified CPGs and would generate a 3′-phosphate after cleavage from CPGs. The procedure for templated chemical ligation of linear DNA strands into cyclic triangles was adapted from the methods reported by Damha and coworkers.^21^ Typically, 3.5 nmol of CTS strand and its corresponding template strand were separately dried and re-dissolved in 250 μL of MES buffer. Then, they were combined and incubated on ice for 30 min. After this, 500 μL of cyanogen bromide solution (5 M in anhydrous acetonitrile) was added, and the reaction mixture was vortexed for 30 min on ice. Then, 40 equivalents of a LiClO_4_ solution (2% w/v in acetone) was added to the reaction mixture, and the reaction mixture was then put on dry ice for 30 min followed by centrifugation at 14 000 rpm for 10 min at 4 °C. The supernatant was discarded, and the residual pellet was obtained, re-dissolved in 1 mL of Milli-Q water and desalted by Sephadex G-25 column chromatography. Then, the desired cyclic products could be separated from their linear analogs by denaturing the PAGE gels.

### TEM

TEM was carried out using Philips Tecnai 12, operated at an acceleration voltage of 120 kV. Typically, 10 μL of the tetrahedron samples were deposited on a slice of Parafilm™, and then, a 200-mesh carbon-coated copper grid was placed on the sample and allowed to adsorb for 45 s. After this, the excess solution was removed with a filter paper, and this copper grid was placed on a drop of 10 μL of Milli-Q water for another 30 s. Then, the grid was roughly dried with filter paper, dried in air for 30 min, and then moved into a desiccator prior to imaging.

### AFM

High-resolution AFM imaging was conducted on HOPG substrates in the solution phase using a BioScope Catalyst Atomic Force Microscope system (Bruker Nano, Santa Barbara, CA) equipped with a piezoelectric AFM scanner to drive the movement of the AFM probe in *Z*-axis with the range of 26 μm. The maximum *X*–*Y* scan range was 150 μm × 150 μm. A silicon nitride AFM tip (SCANASYST-FLUID+, Bruker Nano, Santa Barbara, CA) with a spring constant of 0.7 N m^−1^ was used, and the actual value of the spring constant was calibrated by a thermal tune method prior to imaging. Imaging was then performed in the tapping mode and peak force tapping mode at the scan rate of 1 Hz. The resolution of AFM imaging was selected as 256 × 256. The AFM images were processed and analyzed on AFM offline software, Nano Scope Analysis (v 1.5, Bruker Nano, Santa Barbara, CA). The height images were flattened using 2nd order flattening, and no further image processing was performed. Data processing was performed using GraphPad Prism (v6, GraphPad Software, San Diego, CA).

### Conjugation and purification of gold nanoparticles to DNA strands

The procedure for the preparation of DNA mono-functionalized gold nanoparticles was adapted from the protocol reported by Taton and coworkers.^22^ Typically, 10 mL of citrate-stabilized gold nanoparticles with a mean diameter of 5 nm was mixed with 2 mg of bis(*p*-sulfonatophenyl)phenylphosphine dihydrate dipotassium salt (BSPP) and shaken for over 16 h at low speed at room temperature. Then, the mixture was centrifuged at × 500 g for 30 min, the supernatant was discarded, and the residue precipitate was dissolved in 100 μL of 0.5 mM BSPP solution. After this, 50 μL of methanol was added and mixed thoroughly, and the mixture was centrifuged at ×10 000*g* for 30 min. The supernatant was then discarded, the residue precipitate was dissolved in 100 μL of 0.5 mM BSPP solution, and 11 μL of 5× TBE buffer was added. The BSPP-passivated gold nanoparticles were quantitated by UV absorbance at 520 nm. The disulfide-functionalized DNA strand was first treated with tris (2-carboxyethyl) phosphine (TCEP) for 1 h and then mixed with BSPP-passivated gold nanoparticles. After this, 1 M NaCl solution was added until the final concentration of NaCl reached 0.05 M. Then, the mixture was shaken for 12 h at low speed at room temperature. The DNA mono-functionalized gold nanoparticles were separated from the bare nanoparticles by 3% agarose gel electrophoresis in the 0.5× TBE buffer at a constant voltage of 100 V for 45 min. The desired band was cut off and crushed and incubated in Milli-Q water at 4 °C for 12 h. The extracted gold nanoparticle-DNA conjugates were concentrated with a filter membrane with a molecular weight cut-off of 1000 g mol^−1^.

### Preparation of the DNA tetrahedron (py-THD)

Self-assembly of the designed DNA tetrahedron could be realized *via* two different approaches. In the stepwise approach, the triangular-shaped, cyclic single-stranded DNA building blocks T1, T2 and T3 with an equimolar concentration of 0.6 μM were first hybridized with their respective complementary strands L1, L2 and L3 separately to form the partially double-stranded pairs P1, P2 and P3, respectively. Subsequently, P1, P2 and P3 could be added in a stepwise, order-independent manner to form the final py-THD. In the one-pot approach, all the component DNA strands T1, T2, T3, L1, L2, and L3 with an equimolar concentration of 0.6 μM were randomly mixed together and left for 10 min to form py-THD. Both approaches were performed at room temperature without any annealing process.

### Preparation of the gold nanoparticle-anchored DNA tetrahedron (py-THD-AuNP)

Typically, 4.40 pmoles of each of the ligated triangles T1, T2, and T3 were combined with equimolar amounts of the AuNP-functionalized DNA strands AuNP-L1, AuNP-L2 and AuNP-L3 in a final total volume of 50 μL (with a concentration of 88 pM for each strand) at room temperature to form the desired product. Note that TAMg^2+^ buffer should be avoided because DNA-functionalized gold nanoparticles easily aggregate in Mg^2+^ buffered solutions.

### Cell culture

The three cancer cell lines HeLa, KB and MCF-7 cells were purchased from ATCC. Dulbecco's Modified Eagle Medium (DMEM), fetal bovine serum (FBS), penicillin-streptomycin solution and trypsin containing EDTA were purchased from Thermo Fisher. MCF-7 breast adenocarcinoma cells, human hepatocellular carcinoma cell, or KB cells were routinely cultured in Dulbecco's modified Eagle's medium (DMEM) supplemented with 10% of FBS and 1% of penicillin with streptomycin. The cells were incubated under a humidified 5% CO_2_ atmosphere at 37 °C. When cells grew up to about 95% confluence, they were treated with a standard trypsin-based technique and re-seeded in confocal imaging dishes of concentration about 4 × 10^4^ cell L^−1^. Samples were added to the cells with ∼70% confluence and then incubated for additional 12 h.

### Confocal fluorescence microscopy imaging

KB, HeLa or MCF-7 cells were seeded and cultured overnight in glass bottom dishes. Samples were added and incubated for few hours. After washing with buffers for 3 times, cell imaging was performed. For colocalization studies, 3 μL of 0.5 mM of the relevant tracker was added and incubated with cell samples for ∼30 min. The *λ*_ex_ of Cy3 is 514 nm and the *λ*_em_ region is from 550 to 600 nm, whereas the *λ*_ex_ of Cy5 is 633 nm and the *λ*_em_ region is from 650 to 700 nm. The *λ*_ex_ of hoechst is 405 nm and the *λ*_em_ region is from 430 to 470 nm.

### Fetal bovine serum assay

Herein, 4.1 μM of py-THDs in 1 X TAMg was spiked with fetal bovine serum (FBS) to yield a final composition of 220 ng μL^−1^ DNA in 10% FBS in DMEM followed by incubation at 37 °C for 0–24 h. For denaturing analysis, the sample was denatured by heating at 60 °C for 20 min with 95% formamide. Samples were then characterized by 15% denaturing PAGE, stained with StainAll, and analyzed *via* Image J. The gel analysis resulted in a fingerprint DNA band pattern of each DNA components that could be tracked across time points. Band intensity was obtained with ImageJ, further plotted with respect to time and fitted to first order exponential decay.

### Flow cytometry

Typically, 1 × 10^5^ HeLa cells were seeded on 6-well plates and cultured overnight followed by incubation with Cy5-labeled py-THDs for 12 h. After washing few times with PBS, cells were analyzed by flow cytometry. For the uptake mechanism study, 15 0000 HeLa cells were simply pre-treated with either 0.45 M sucrose, 0.08 M NaN_3_, 200 μM genistein at 37 °C or only cultured cells at 4 °C. Then, 33 nM of Cy5-labeled py-THDs were added and incubated for 3 h. The cells were then trypsinized for flow cytometric analysis. The fluorescence intensity in only 33 nM py-THD-treated cells was set as a control. The Cy5 fluorescence signal was excited at a wavelength of 633 nm and obtained from 640 to 680 nm.

## Results and discussion

**Fig. 1:**
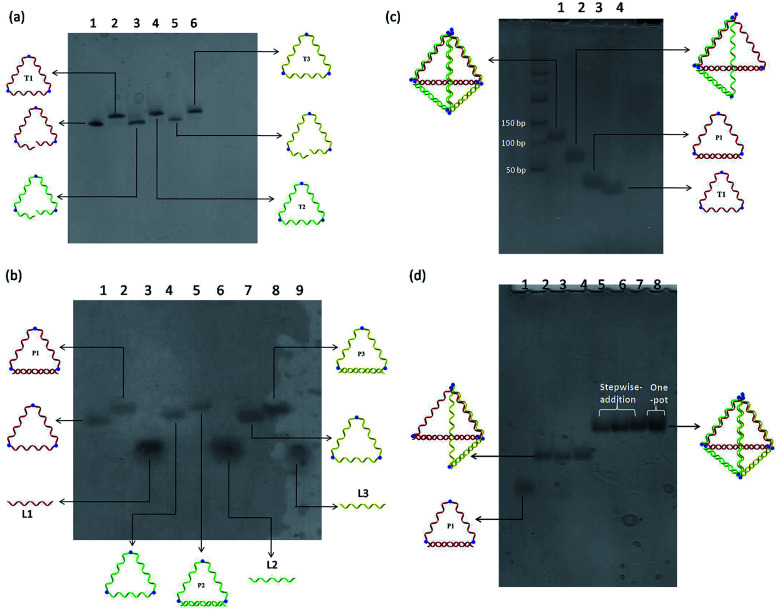
(a) Denaturing PAGE analysis of the pure ligated cyclic triangles compared with their open-ended DNA strands. (b) Native PAGE analysis of the hybridization of cyclic triangles with their complementary strands. (c) Native PAGE analysis of the stepwise formation of DNA tetrahedron. (d) Native PAGE analysis of the formation of DNA tetrahedron with different orders of strand addition. Lane 1: P1; Lane 2: P1 + P2; Lane 3: P1 + P3; Lane 4: P2 + P3; Lane 5: P1 + P2 + P3; Lane 6: P2 + P3 + P1; Lane 7: P3 + P1 + P2; Lane 8: One-pot mixing.

A modular self-assembly strategy was used to create a DNA tetrahedron with six 15-base pair (bp) edges connected at four vertices. In our design, initial efforts were focused on the generation of three triangular-shaped, cyclic single-stranded DNA building blocks. To finely control their geometry, we followed Sleiman's approach to insert small organic vertex molecules as rigid junctions.^23^ This would allow to control the angle between two single-stranded DNA fragments. Moreover, three 45-mer DNA strands (CTS1, CTS2 and CTS3) embedded with three terphenyl molecules at the position 8, 24 and 40 were chemically synthesized on 3′-phosphate-functionalized control pore glasses (CPGs). The two open ends of the three CTS strands were hybridized to their corresponding complementary DNA strands L1, L2, and L3 followed by a chemical ligation reaction using cyanogen bromide to generate three closed and cyclic triangular-shaped DNA building blocks (T1, T2 and T3) with yields ranging from 25% to 35%.^21^ These cyclic products were purified and characterized by denaturing PAGE (Fig. 1a) and MALDI-TOF mass spectroscopy analyses (Fig. S1†). It is important to emphasize that the presence of organic molecules does not reduce the biocompatibility in a biological system as shown by Sleiman's group.^24^ Initially, native PAGE analysis was used to evaluate the stepwise addition of the three chemically ligated DNA strands followed by the addition of three 15-mer complementary strands L1, L2 and L3 to form the pyramidal DNA nanostructure. However, no clear DNA band shifting can be observed after the addition of the three T strands. It is difficult to know whether the designed DNA tetrahedron is successfully formed. We believe that this failure may be due to the entropy loss and rotation hindrance arising from the linear-ladder like hybridization among the three closed triangle strands. To synergistically facilitate the formation of the thermodynamically favorable DNA tetrahedron structure, at first, L1, L2 and L3 were added and hybridized to one side of the corresponding T1, T2 and T3, respectively (Fig. 1b). Afterwards, native PAGE analysis confirmed the stepwise addition of the three pairs DNA building blocks (P1 to P3) to form a discrete pyramidal DNA tetrahedron (py-THD) at room temperature within 10 min (Scheme 1 and Fig. 1c).

Moreover, DNA ladders were used to quantitatively estimate the molecular size of the resulting self-assembled structures. It was found that the molecular mass of the final structures with ∼a bit larger than 100 base pairs was in good consistency with our design comprising 90 base pairs and nine vertex molecules. DNA bands with the same mobility were obtained regardless of the order of the addition of the DNA building blocks (P1, P2 and P3) (Fig. 1d). These resulting clean and single bands in each lane indicated the perfectly formed intermediate products and final tetrahedron structure in a 100% yield. This was encouraging as DNA tetrahedra assembled by other approaches usually suffer from the uncontrolled formation of other undefined species.^8^ Interestingly, this py-THD can also be assembled in a one-pot reaction with quantitative yield by direct mixing of T1, T2, T3, L1, L2 and L3 strands at room temperature (lane 8 in Fig. 1d). Based on these two different assembling strategies, we concluded that the formation of py-THD was highly feasible without the thermal annealing process. In our design, the regular tetrahedron with hollow faces consists of double-helical edges at the bottom, whereas the rest of the top three edges are in a linear-ladder arrangement; this means that two complementary DNA strands form a “ladder pair” through π–π stacking interaction from the face-to-face of the pyrimidine/purine rings of nucleotides along the DNA strands. Additionally, these “ladder pairs” assemble through hydrogen bonds between the complementary nitrogen base pairs of adenine and thymine (A–T) or guanine and cytosine (G–C) and packed along the horizontal axis. No coiling between the DNA strands occur in this ladder-like DNA structure.

**Fig. 2:**
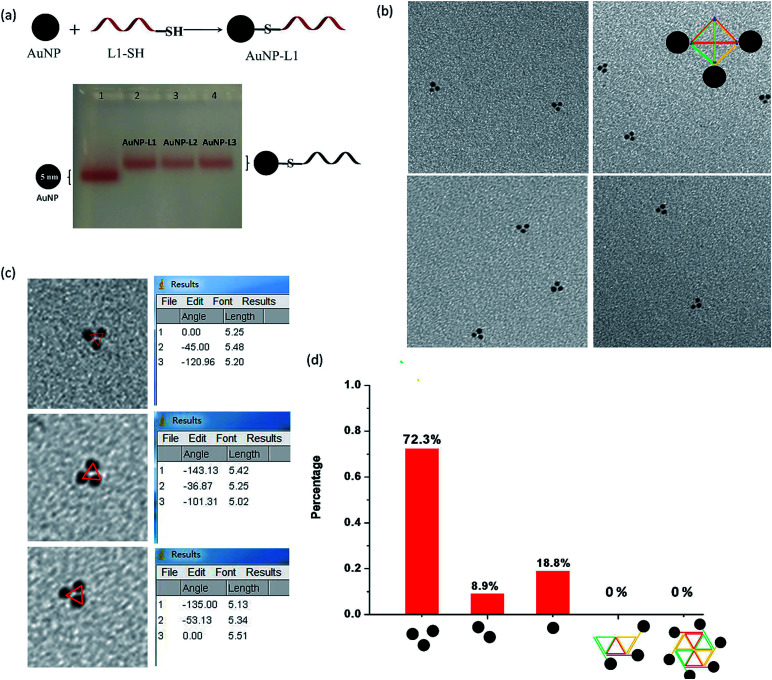
(a) Schematic of the formation of AuNP-DNA conjugates *via* thiol chemistry and agarose gel analysis of isolated, purified AuNP-DNA conjugates. (b) Representative TEM images of AuNP-tethered DNA tetrahedron. (c) Measured interparticle distance of gold nanoparticles tethered on DNA tetrahedron, data analyzed by ImageJ. (d) Statistical distribution analysis of the patterns of gold nanoparticles found in TEM studies, *N* = 172.

Each L strand has its unique sequence in our design, and it is highly possible to specifically position different types of substances at the three vertexes of py-THD *via* a wide range of chemical conjugation approaches. To demonstrate the capability to construct discrete DNA nanostructures decorated with metallic nanoparticles based on this newly designed tetrahedron, citrate-passivated 5 nm gold nanoparticles (AuNPs) were first conjugated to 3′-end thiol-modified DNA strands (L1-SH to L3-SH) through typical thiol chemistry.^25^ The number of the DNA strand per gold nanoparticle was quantified based on a previously reported UV-visible spectroscopy-based quantification method (Fig. S2†).^26^ The AuNP-DNA mono-conjugates (*e.g.* AuNP-L1, AuNP-L2 and AuNP-L3) were purified and analysed by 3% agarose gel electrophoresis (AGE) separation (Fig. 2a and S3†). By utilizing these AuNP-DNA mono-conjugates for self-assembly, the DNA tetrahedron anchored with three gold nanoparticles (py-THD-AuNP) at one facet was formed. Above 70% of AuNPs are in the pattern of a triangle with an average interparticle distance of 5.28 ± 0.05 nm, as characterized by TEM imaging (Fig. 2b–d). Statistical analyses of the patterning of AuNPs strongly suggested that a 3D tetrahedron was assembled instead of a 2D structure.

Conceptually, a 2D structure would provide different patterns of gold nanoparticles, as shown in Fig. 2d, but no such patterning of AuNPs was found. Thus, the DNA tetrahedron tethered with three gold nanoparticles at the same facet dominated in the resulting products. Additionally, the measured interparticle distance indicates that the three AuNPs are in a very close proximity in the 3D architecture; this is highly attributed to the presence of flexible DNA spacers between the edges of py-THD and AuNPs. Moreover, both the 3′- and 5′-ends of the L strands could easily be modified with additional functional linkers for further conjugation of targeting ligands, fluorescence probes, and therapeutic agents^27–29^ for the construction of DNA tetrahedral nanocarriers.^30^ These L strands could also be designed to have single-stranded DNAs as the sticky ends for the further fabrication of much larger and more complex assemblies using the 3D DNA tetrahedron as the building blocks.^31^

**Fig. 3:**
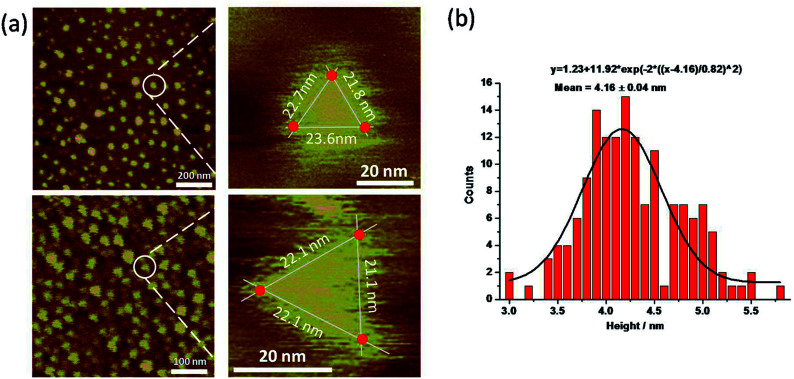
(a) Representative AFM images of py-THD immobilized onto HOPG substrates. Zoom-in AFM images of individual particle with triangular-shaped. (b) Statistical height analysis of AFM images for py-THD (*N* = 152).

The py-THD structure was also imaged by liquid AFM. AFM images showed populations of individual DNA particles (Fig. 3a and S4†). In the enlarged images, the particle resembled the designed tetrahedral structure with its three edges measured, and the center region was notably higher than the peripheral part. Note that longer apparent lateral measurements were usually obtained in the magnified images due to the convolution of the AFM tip. Statistical analyses of individual particles in AFM height images showed an average measured height of ∼4.16 ± 0.04 nm (Fig. 3b), which was highly consistent with the theoretical height of the designed py-THD. No such discrete particle feature was observed in the AFM studies in the control samples, which only consisted of PBS buffer solution. Till now, these findings provided direct evidence that it was highly feasible to design and self-assemble DNA nanostructures without fully double helical formation. This tetrahedron made of DNA could potentially facilitate the loading of very small therapeutic agents, with a volume of ∼4.7 nm^3^, such as small protein, enzymes, or molecular drugs as compared to all the previously reported DNA tetrahedron.^12,32–34^

**Fig. 4:**
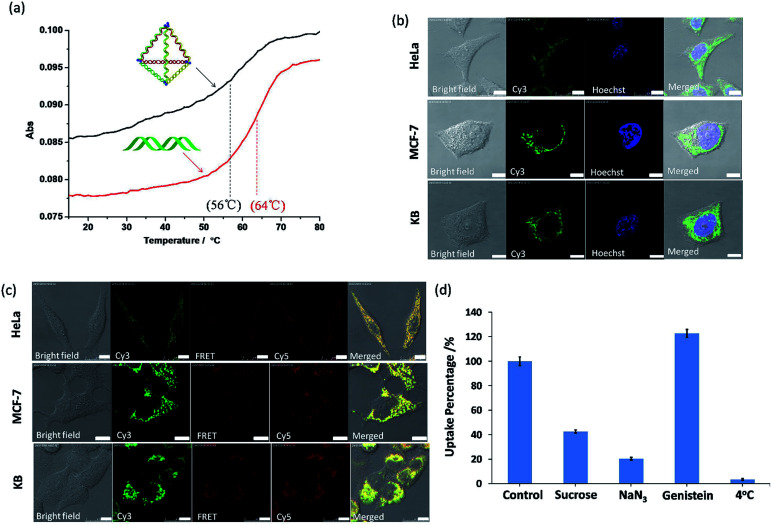
(a) Thermal denaturation studies of py-THDs and its duplexes. (b) Confocal fluorescence images of Cy3-labeled py-THDs incubated with HeLa, MCF-7 and KB cells for 12 h. (c) FRET studies of doubly Cy-labeled py-THDs in different cell lines. Samples were excited with 514 nm laser and their emission were collected from 550 to 600 nm (green channel for Cy3) and 650 to 700 nm (red channel for Cy5) (d) flow cytometry studies showing the effects of inhibitors on cellular uptake of py-THDs. Scale bar is 10 μm.

To consider these very small py-THDs as nanocarriers for targeted drug delivery, their thermal and biological stability are the key concerns. Thermal denaturation studies showed that the melting temperature (Tm) value for the self-assembled py-THD was found to be ∼56 °C (Fig. 4a). This suggests that py-THD could maintain its structural integrity at the biological temperature (∼37 °C); thus, it is an important support for applications in cellular environment. However, py-THD showed a much lower Tm than its individual standard B-form DNA duplexes with the same sequences and lengths added up all together. This thermal retardation is possibly attributed to the linear-ladder arrangement of the formation of DNA duplexes at the top edges of py-THD.^35^

To further explore its biological stability, this py-THD was subjected to 10% fetal bovine serum (FBS) assay and analyzed by denaturing PAGE (Fig. S5†). An extended half-life of 3.95 h has been obtained when compared with that of the duplex DNA strands that easily degrade within 15 min.^36^ These results suggested that this newly designed DNA tetrahedron structure consisting of three fully ligated DNA strands could provide extra protections for enzymatic degradation. In fact, this is not unexpected as several studies have reported that fully ligated DNA strands can confer DNA nanostructures with enhanced cellular stability.^37^ Moreover, py-THD can be easily taken up by the most common cell lines such as HeLa, MF-7 and KB cells, as confirmed by confocal fluorescence microscopy studies (Fig. 4b). Furthermore, this py-THD could be easily functionalized with both Cy3 and Cy5-fluorophores to study its structural integrity in both cells and buffer solutions by fluorescence resonance energy transfer (FRET) experiments. We found that py-THD could enter cells as a cohesive, and its structure remained intact after 24 h (Fig. 4c). These cellular FRET results are also consistent with those obtained under buffer conditions, resulting in strong FRET signals at 662 nm (Fig. S6†). As compared to the reported DNA tetrahedron structures with a larger size, this py-THD can be efficiently taken up by cancer cells after a few h (Fig. S7†). All the abovementioned findings suggested that this novel designed DNA tetrahedron could be biocompatible enough to perform multi-tasks such as tracking,^38^ bioimaging,^39^logic sensing,^16^ detection^40,41^ and/or drug delivery^42^ in biological systems.

To explore the cellular uptake mechanism of these py-THDs, they were incubated with different endocytosis inhibitors in HeLa cells, and flow cytometry studies were used for analysis. As shown in Fig. 4d, the internalization of py-THDs in cells exhibited a substantial decrease at 4 °C. This significant drop of the uptake is possibly due to the presence of a rigid cell membrane at this low temperature, resulting in improper translocation *via* physicochemical interaction between the cell membranes and py-THDs.^43^ Additionally, the cellular uptake of py-THDs is inhibited by 80% after treatment with sodium azide (NaN_3_), which is used to disturb the ATP production and block the endocytosis pathway.^44,45^ Pre-treatment of HeLa cells with genistein before incubation with py-THDs showed no significant change in their uptake.^46^ On the other hand, pre-treatment with sucrose, which inhibits the formation of clathrin-coated vesicles, also shows a significant decrease in the uptake of py-THDs.^47^ These results clearly indicate that py-THDs uptake by living cells occurs through a temperature-, energy-dependent and clathrin-mediated endocytosis pathway.

## Conclusions

In summary, we have successfully assembled a discrete DNA tetrahedron in unconventional ladder-like arrangements at room temperature either *via* a modular or one-pot approach. This strategy fosters the formation of the dominant, thermodynamically stable tetrahedral product in a quantitative yield but does not require a DNA trigger or thermal annealing treatment to initiate the self-assembly. They exhibit extra protections for enzymatic degradation and substantial cellular uptake in different cell lines *via* temperature-, energy-dependent and clathrin-mediated endocytosis pathway. This small tetrahedron was made of DNA that could facilitate the loading of very small and temperature-sensitive cargos with volume of ∼4.7 nm^3^ for targeted delivery applications. Much larger and highly ordered DNA assemblies could also be further fabricated using this newly formed DNA tetrahedron with sticky ends as the building blocks. This novel assembly strategy developed for DNA tetrahedral could potentially be extended to other highly complex polyhedra; this indicated its generalizability.

## Conflicts of interest

There are no conflicts to declare.

## Supplementary Material

NA-001-C8NA00323H-s001
