# Supplementary material for: Facile construction of a DNA tetrahedron in unconventional ladder-like arrangements at room temperature
Source: Nanoscale Adv. 2018 Dec 27;1(3):1240–8. doi: 10.1039/c8na00323h (PMC9473169; doi:10.1039/c8na00323h)
Supplement: NA-001-C8NA00323H-s001 [file NA-001-C8NA00323H-s001.pdf]

Electronic Supplementary Material (ESI) for Nanoscale Advances.
This journal is © The Royal Society of Chemistry 2018

Facile Construction of DNA Tetrahedron in Unconventional Ladder-Like Arrangements at Room Temperature

Ziwen Dai,[a] Hoi Man Leung,[a] Qi Gao,[b] Fei Wang,[a] Sze Wing Wong,[a] Ling Sum Liu,[a] Yu Ju Au,[a] King Wai Chiu Lai,[b]* and Pik Kwan Lo[a,c]*

[a] Department of Chemistry, [b] Department of Biomedical Engineering, City University of Hong Kong, Tat Chee Avenue, Kowloon Tong, Hong Kong SAR

[c] Key Laboratory of Biochip Technology, Biotech and Health Care, Shenzhen Research Institute of City University of Hong Kong, Shenzhen 518057, China

Materials

Acetic acid, urea, boric acid, ethylenediaminetetraacetic acid disodium salt dihydrate (EDTA), formamide, magnesium chloride hexahydrate, StainsAll®, tris(hydroxymethyl)aminomethane (Tris), *N,N,N',N'*-tetramethylethylenediamine, acrylamide, bis-acrylamide, ammonium persulfate and glycerol were used as purchased from Sigma Aldrich. 1000Å nucleoside-derivatized LCAA-CPG solid supports with loading densities of 25-40 μmol/g, reagents used for automated DNA synthesis were purchased from BioAutomation. Sephadex G-25 (super fine DNA grade) was used as purchased from Amersham Biosciences. 1 X TA-Mg^{2+} buffer was composed of 45 mM Tris, 7.6 mM $MgCl_2$, with pH adjusted to 8.0 using glacial acetic acid. 1 X TBE buffer was composed of 90 mM Tris and boric acid, 1.1 mM EDTA, with a pH of ~ 8. Gold nanoparticles with a mean diameter of 5 nm and stabilized in citrate buffer, Bis(p-sulfonatophenyl)phenylphosphine dihydrate dipotassium salt (BSPP) and tris(2-carboxyethyl)phosphine (TCEP) were purchased from Sigma Aldrich. Cy3 or Cy5-labeled DNAs were purchased from Sangon Biotech Shanghai. All other regular DNA strands were purchased from Integrated DNA Technologies.

Instrumentation

Fluorescence measurements were conducted on HORIBA JobinYvon™ FluoroMax-4 spectrofluorometer. Standard solid-phase oligonucleotide synthesis was performed on BioAutomation MerMade MM6 DNA synthesizer. UV-Vis measurements were carried out on NanoDrop 1000 spectrophotometer. PAGE Gel electrophoresis experiments were carried out on a polyacrylamide 20 X 20 cm Maxi Vertical electrophoresis apparatus (MV-20DSYS). Agarose gel electrophoresis was performed on Bio-Rad Mini-Sub Cell GT Cell Apparatus with a 7 X 7 cm gel tray. Thermal denaturation was performed on Cary 300 UV-Vis Spectrophotometer. Matrix-Assisted Laser Deposition-Ionization Time-of Flight Mass Spectrometry (MALDI-TOF MS) was performed on Bruker Autoflex Mass Spectrometer.

DNA sequences

Table S1. Sequences of DNA strands for the construction of DNA tetrahedron

	Sequence (5'→3')	ε_{260} [L/(mole·cm)]
CTS1	TC GGT AGA V GAT AGC CCA GAT CGG V AGA GTA ATC TCT TGA V TGA GCA C	450200
CTS2	TA CCA CTA V CCT GAC CGA TTG GAC V CCG ATC TGG GCT ATC V CCT CAC A	414500
CTS3	TC CTA CCT V TCA AGA GAT TAC TCT V GTC CAA TCG GTC AGG V TGT CGT C	419600
L1	TCT ACC GAG TGC TCA	139600
L1-SH	(Thiol) TAT TCT ACC GAG TGC TCA ATA GAA ACT AAA AGA ATG ATG ATA CAT TAT AGA TAC TAC ACT TAT	644700
L1-Cy3	(Cy3) TTC TAC CGA GTG CTC A	147700
L2	TAG TGG TAT GTG AGG	155000
L2-SH	(Thiol) TTA TAG TGG TAT GTG AGG ATA GAA ACT AAA AGA ATG ATG ATA CAT TAT AGA TAC TAC ACT TAT	661800
L3	AGG TAG GAG ACG ACA	164700
L3-SH	(Thiol) ATT AGG TAG GAG ACG ACA ATA GAA ACT AAA AGA ATG ATG ATA CAT TAT AGA TAC TAC ACT TAT	669800
L3-Cy5	AGG TAG GAG ACG ACA T (Cy5)	172100

(a) General Procedures for Solid-Phase DNA Synthesis:

DNA synthesis was performed on 1 μmole scale, starting from the required nucleotide modified 1000 Å LCAA-CPG solid support. The vertex molecule, 2-cyanoethyl (4''-(trityloxy)-[1,1':3',1''-terphenyl]-4-yl) diisopropylphosphoramidite was site-specifically coupled onto the growing oligonucleotide chain with a prolonged coupling time of 10 min and 3 couplings. The coupling efficiency was monitored by the removed trityl concentration level. All sequences were fully deprotected in concentrated ammonium hydroxide solution at 55°C for over 8 h.

(b) Purification:

Crude DNA strands were purified on 15% polyacrylamide/8M urea polyacrylamide gels at a constant current of 30 mA for 2 h (0.5 h at 250V followed by 1.5 h at 500 V), using 1 X TBE buffer. After electrophoresis, the plates were wrapped in plastic film and placed on a fluorescent TLC plate and then illuminated with a UV lamp at 254 nm. The bands were excised quickly and the selected gel pieces were crushed and incubated in 12 mL of sterile water at 55 °C for over 12 h. DNA samples were concentrated to about 1mL, desalted using Sephadex G-25 column chromatography. Quantification is carried by UV/Vis analysis.

(a)

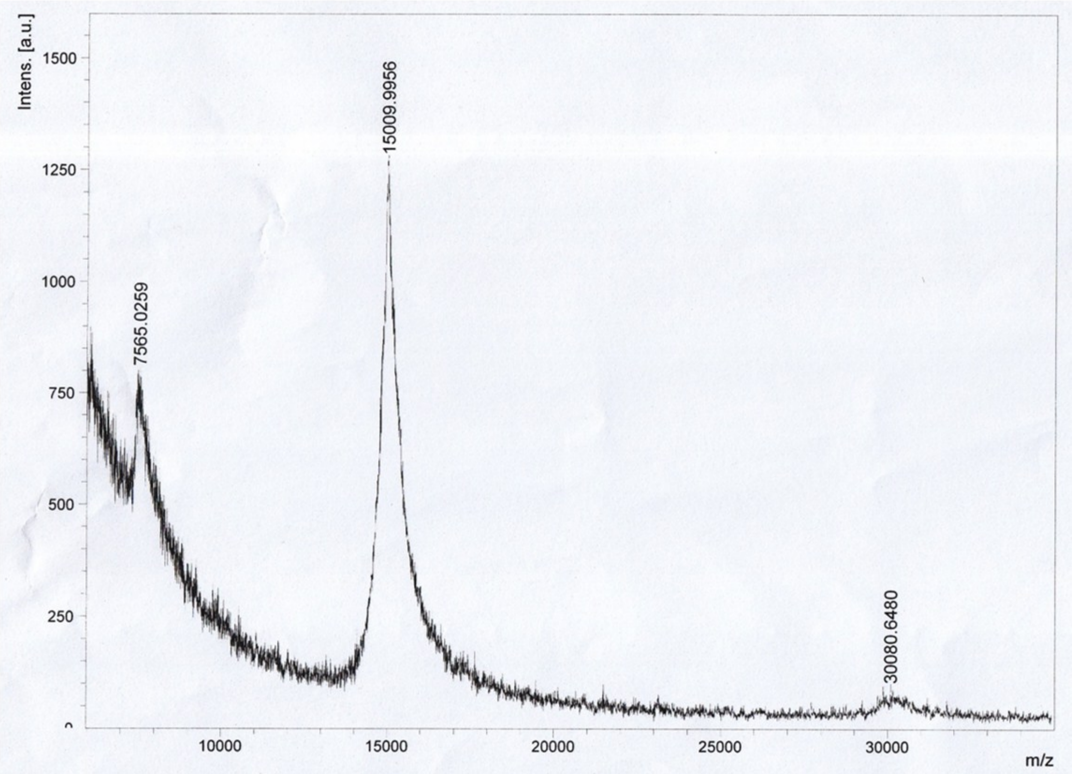
Intens. [a.u.]
1500
1250
1000
750
500
250
0
15009.9956
7565.0259
30080.6480
10000
15000
20000
25000
30000
m/z

(b)

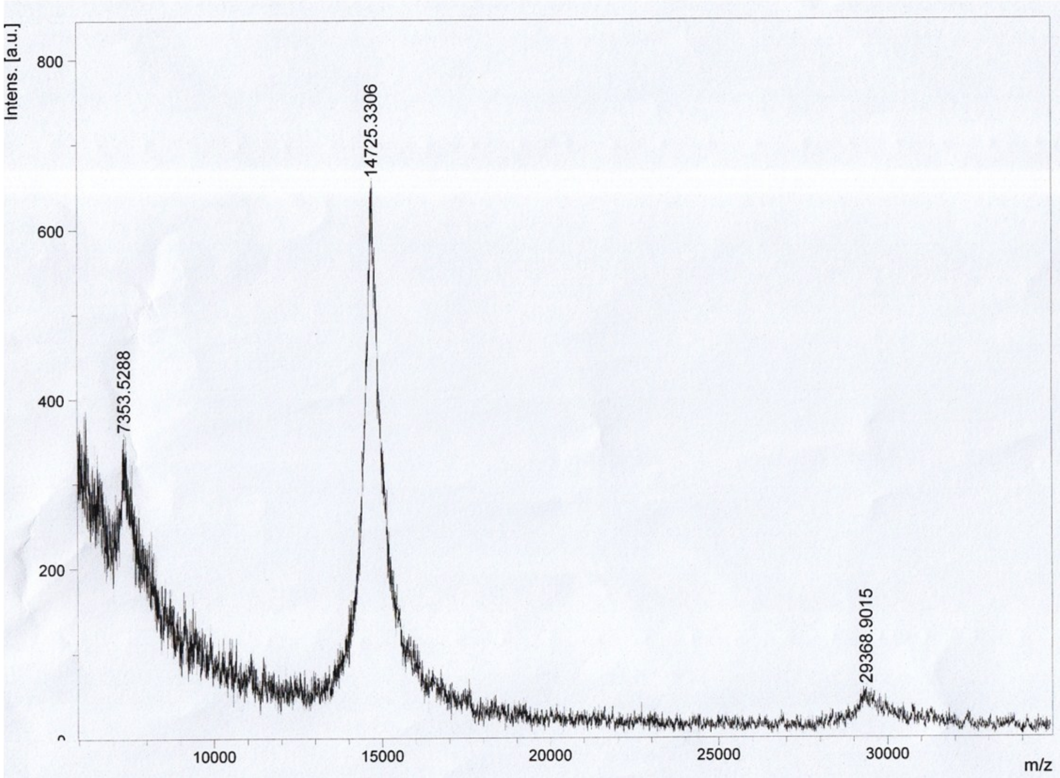
Intens. [a.u.]
800
600
400
200
0
14725.3306
7353.5288
29368.9015
10000
15000
20000
25000
30000
m/z

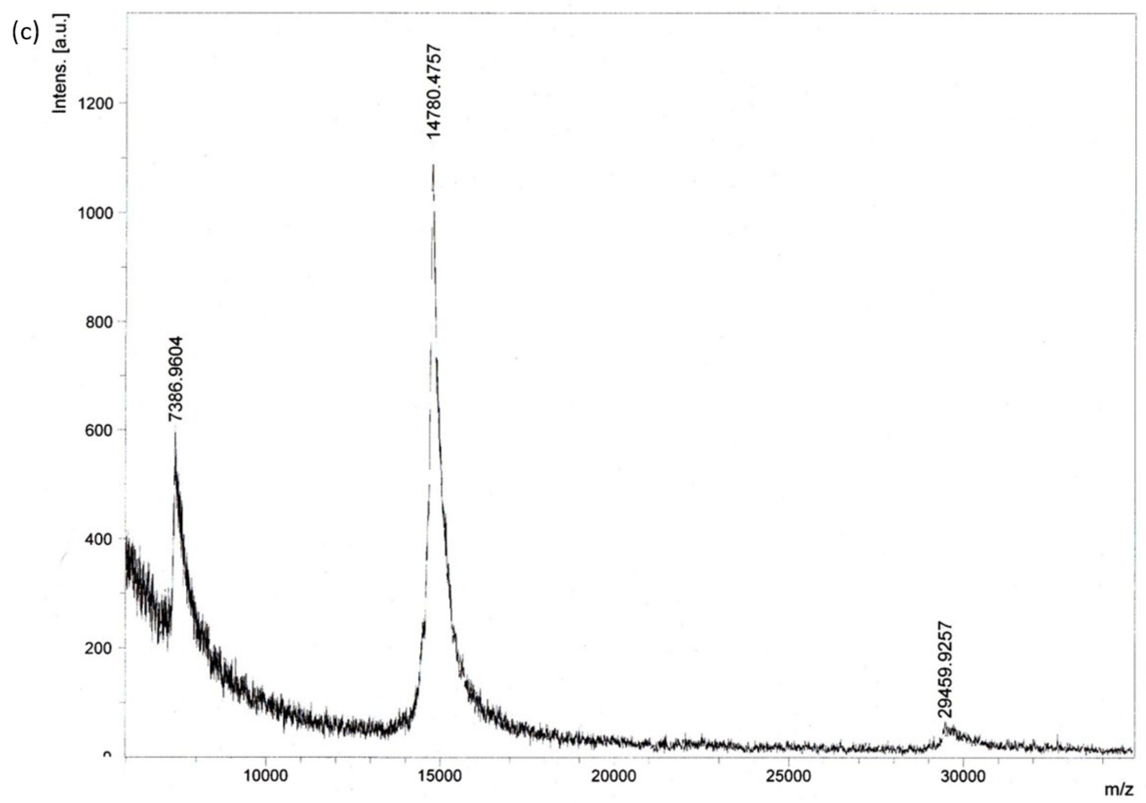

Figure S1 MALDI-TOF spectrum for (a) T1 of m/z calcd 14968.87 g/mol; found, 15009.9956 g/mol $[M]^+$, (b) T2 of m/z calcd 14696.67 g/mol; found, 14725.3306 g/mol $[M]^+$, and (c) T3 of m/z calcd 14772.67 g/mol; found, 14780.4757 g/mol$[M]^+$

The appearance melting temperature (T_m) assay

The appearance melting temperature T_m of DNA tetrahedron was determined with thermal denaturation experiment performed on Cary 300 UV-Vis spectrophotometer. The DNA tetrahedron sample was formed with 4.40 μM of each of the component strand T1, T2, T3, L1, L2 and L3 in 1 X PBS buffer was heated at 1 °C/min from 15 °C to 80 °C with a 1 °C interval and hold for 1 min at each degree. This experiment was performed twice. The appearance melting temperature $\mathbf{T_m}$ was calculated by taking the first derivative of the melting curve.

Quantitation of gold nanoparticle-DNA monoconjugates

To confirm the band excised from the agarose gel containing only one DNA strand per AuNP, we conducted additional experiments to quantify the unlabeled DNA strand bound to gold nanoparticle by

measuring the UV absorbance at 260 nm and at 520 nm. The coverage quantification was based on a UV-visible spectroscopy method reported by Hutchison and coworkers[1] and performed on NanoDrop 1000 spectrophotometer. KCN treatment is used to decompose the purified AuNP-L1, conjugates in order to eliminate the UV/vis absorbance at 260 nm from AuNP core which can interfere with the UV absorbance of DNA at 260 nm. Take AuNP-L1 as an example.

$A260$ dAuNP-L1 = $A260$ L1 + $A260$ dAuNP equation (1)

$A260$ L1 = $A260$ dAuNP-L1 - $A260$ dAuNP equation (2)

Firstly, to determine the concentration of AuNP in AuNP-L1 sample, the UV spectrum of AuNP-L1 conjugate before KCN treatment was measured with absorbance 0.227 at 520 nm, which corresponded to 2.27×10^{-7} mol/L. Next, AuNP-L1 was treated with potassium cyanide (KCN) solution at pH 12 for 8 h to decompose into $KAu(CN)_2$ and L1 strands. The absorbance of this decomposed mixture ($A260$ dAuNP-L1) was measured at 260 nm and is found to be 0.073 which is contributed by both L1 strands and the decomposed AuNPs (equation 1).

Next, to determine the absorbance of AuNP core contributed at 260 nm after KCN treatment, calibration curve of the decomposed AuNPs was obtained. As we have performed identical operations in our previous research work with the same gold nanoparticles.[2] we used the previously obtained calibration curve directly. From the decomposed AuNP absorbance-concentration calibration curve, the absorbance of AuNP core contributed at 260 nm:

$A260dAuNP = 0.0269 + (2.27\times10^{-7})* 507500 =0.1421$

From equation 2, the absorbance contributed by L1 at 260 nm:

$A260L1 = 0.227-0.1421 = 0.0849$

Meanwhile, different concentrations of SCS1-SH strand were prepared and their absorbance at 260 nm were also measured and plotted against corresponding DNA concentrations. A good linear regression of the calibration curve was also obtained. From the SCS1 absorbance-concentration calibration curve, we could get the DNA concentration:

$[L1\text{-}SH] = (0.0849-0.0003063)/41250 = 1.43 \times 10^{-7}$ mol/L

Then the number of DNA strand per gold nanoparticle:

$N = (1.43 \times 2 \times 10^{-7}) / (32.27 \times 10^{-7}) = 1.17$

The same procedure was performed to confirm that we have also obtained the DNA-AuNP mono-conjugate for both L2-SH and L3-SH. We found that the number of DNA strand per gold nanoparticle for L2-SH and L3-SH was 1.38 and 1.42 respectively.

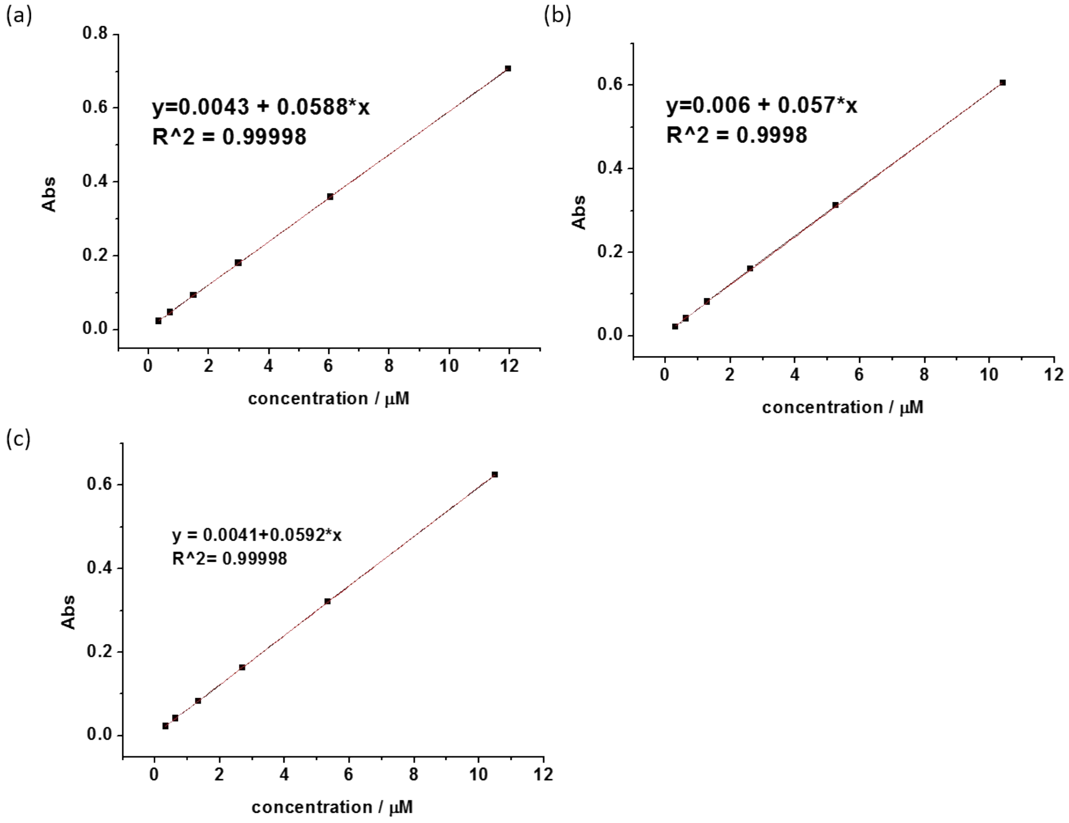

Figure S2. Calibration curve for (a) L1-SH, (b) L2-SH and (c) L3-SH.

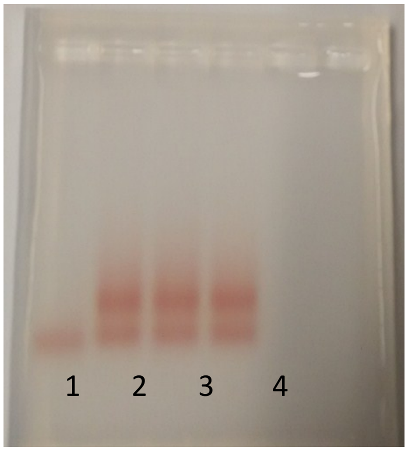

Figure S3. Agarose gel electrophoresis analysis of the formation of AuNP-DNA monoconjugate. Lane 1: bare AuNPs; Lane 2-4: reaction mixture of AuNPs with L1-SH, L2-SH and L3-SH respectively.

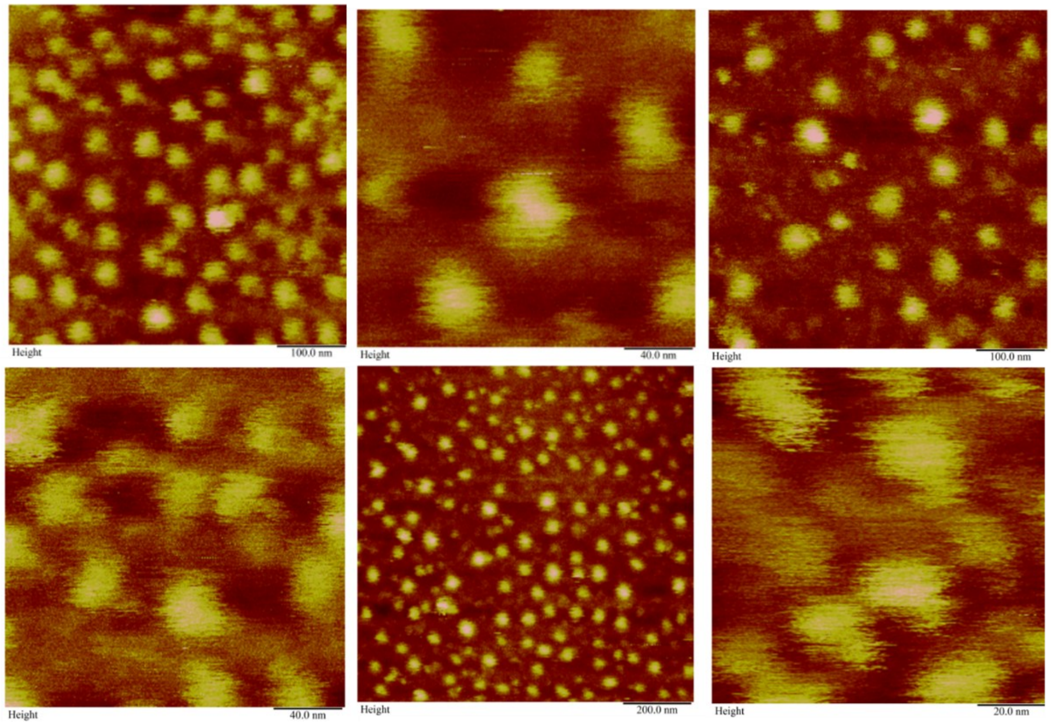

Figure S4. Liquid AFM images of py-THDs.

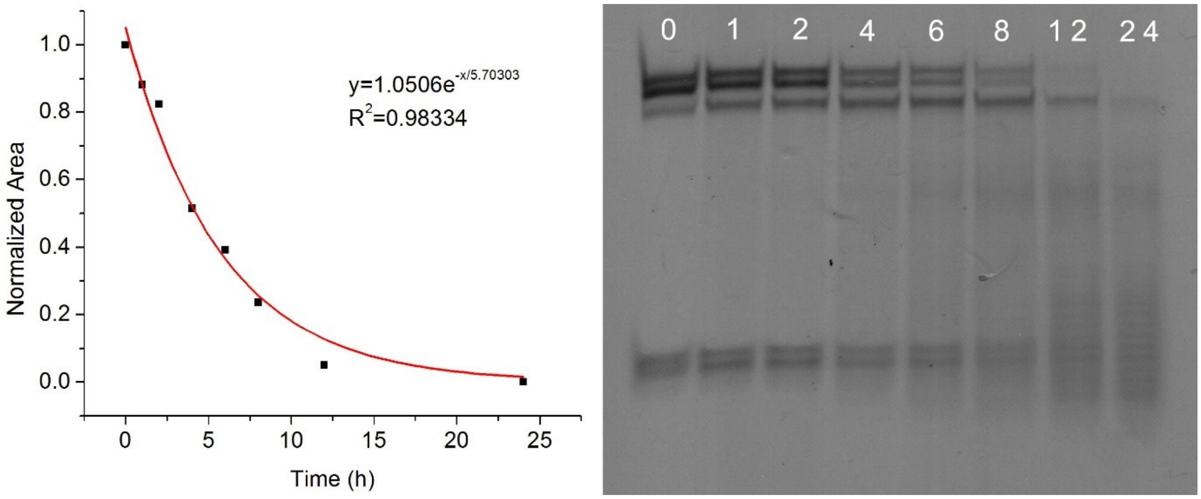

Figure S5. Denaturing PAGE analysis of py-THD in FBS digestion test under different time points.

To determine the mean half-life, bands at 0 time point were identified and used as baseline to remove the background intensity. The decay rate (λ) and half-life ($t_{1/2}$) were derived as follows.

$$I = I_o e^{-\lambda t}$$

$$\tau = \frac{1}{\lambda},$$

$$t_{1/2} = \tau In2$$

$= 3.95$ h

where $\tau$is time constant, I_0 is the initial band intensity and I is the band intensity at time t.

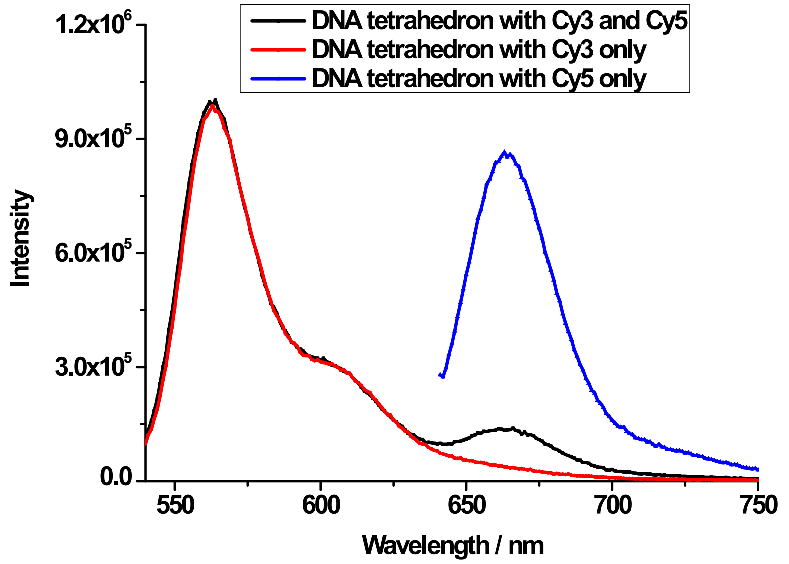

Figure S6. The FRET analysis of Cy-labeled DNA tetrahedron in solution.

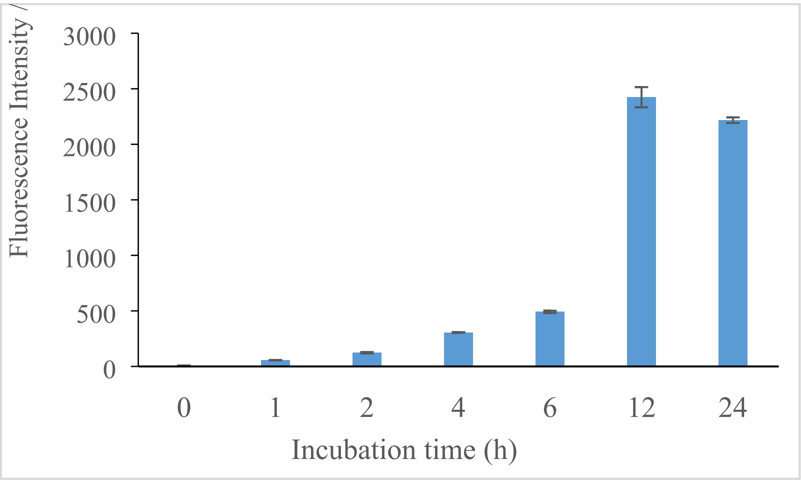

Figure S7. Flow cytometry analysis of the cellular uptake of py-THDs in HeLa cells.

Theoretical height calculation

To facilitate the analysis of the physical parameters obtained from characterization on the DNA tetrahedron, it is necessary to discuss and clarify some geometrical parameters associated with standard tetrahedron model. This DNA tetrahedron constructed by our strategy could be considered as a standard tetrahedron with some negligible deviations. The edge length of the out-of-plane three edges might be slightly different from that of three edges of the bottom facet because the former edges took a ladder-like linear conformation while the latter edges are of standard B-form helix. To simplify data analysis, this DNA tetrahedron was treated as standard model with all edges identical in length.

Thus, if a standard tetrahedron has an edge length of L, then the height H could be calculated by **equation 3**:

$H = L*(\sqrt{6}/3)$ ………………………………………………………………… equation (3)

In our design, the edges of the tetrahedron consisted of 15 base pairs, thus, the edge length of the DNA tetrahedron should be around 5.1 nm. Therefore, the height H could be calculated as

$H= 5.1 *(\sqrt{6}/3) = 4.16$ nm.

Reference

(1) Baldock, B. L.; Hutchison, J. E. UV–Visible Spectroscopy-Based Quantification of Unlabeled DNA Bound to Gold Nanoparticles. *Analytical Chemistry* **2016**, *88* (24), 12072–12080.

(2) Dai, Z.; Lo, P. K. Photo-Switchable Patterning of Gold Nanoparticles along 3D DNA Nanotubes. *Nanoscale* **2018**, *10* (12), 5431–5435.
